# Supplementary material for: An Unusual Case of Listeria monocytogenes-Associated Rhombencephalitis Complicated by Brain Abscesses in Italy, 2024
Source: Infect Dis Rep. 2026 Jan 4;18(1):5. doi: 10.3390/idr18010005 (PMC12821487; doi:10.3390/idr18010005)
Supplement: Supplementary file 1 [file idr-18-00005-s001.zip › idr-4014419-supplementary.pdf]

CARE case report guidelines

CARE Checklist of information to include when writing a case report

(cc) BY-NC-ND

Topic	Item	Checklist item description	Reported on Line
Title	1	The diagnosis or intervention of primary focus followed by the words "case report"	2-3
Key Words	2	2 to 5 key words that identify diagnoses or interventions in this case report, including "case report"	49-52; 146-148
Abstract (no references)	3a	Introduction: What is unique about this case and what does it add to the scientific literature?	47-52;159-164
	3b	Main symptoms and/or important clinical findings	54-57
	3c	The main diagnoses, therapeutic interventions, and outcomes	72-74;103-105
	3d	Conclusion—What is the main "take-away" lesson(s) from this case?	184-196
Introduction	4	One or two paragraphs summarizing why this case is unique (**may include references**)	47-52; 184-196
Patient Information	5a	De-identified patient specific information.	54-60
	5b	Primary concerns and symptoms of the patient.	54-57
	5c	Medical, family, and psycho-social history including relevant genetic information	58-60
	5d	Relevant past interventions with outcomes	58-60
Clinical Findings	6	Describe significant physical examination (PE) and important clinical findings.	64-69
Timeline	7	Historical and current information from this episode of care organized as a timeline	54, 56, 70, 94, 103, 114, 121, 132
Diagnostic Assessment	8a	Diagnostic testing (such as PE, laboratory testing, imaging, surveys).	64-75
	8b	Diagnostic challenges (such as access to testing, financial, or cultural)	98-101
	8c	Diagnosis (including other diagnoses considered)	61-75; 102-105
	8d	Prognosis (such as staging in oncology) where applicable	121-132
Therapeutic Intervention	9a	Types of therapeutic intervention (such as pharmacologic, surgical, preventive, self-care)	64-75; 94-97; 116; 126-131
	9b	Administration of therapeutic intervention (such as dosage, strength, duration)	94-97; 116; 126-131
	9c	Changes in therapeutic intervention (with rationale)	93-97
Follow-up and Outcomes	10a	Clinician and patient-assessed outcomes (if available)	132
	10b	Important follow-up diagnostic and other test results	102-110; 133-134
	10c	Intervention adherence and tolerability (How was this assessed?)	93-115
	10d	Adverse and unanticipated events	121-132
Discussion	11a	A scientific discussion of the strengths AND limitations associated with this case report	146-192
	11b	Discussion of the relevant medical literature **with references**.	146-180
	11c	The scientific rationale for any conclusions (including assessment of possible causes)	177-192
	11d	The primary "take-away" lessons of this case report (without references) in a one paragraph conclusion	193-196
Patient Perspective	12	The patient should share their perspective in one to two paragraphs on the treatment(s) they received	Not applicable
Informed Consent	13	Did the patient give informed consent? Please provide if requested	**Yes** ☐ **No** ☒
